# Antibacterial Effects of *Ramulus mori* Oligosaccharides against *Streptococcus mutans*

**DOI:** 10.3390/foods12173182

**Published:** 2023-08-24

**Authors:** Erna Li, Shipei Li, Siyuan Wang, Qian Li, Daorui Pang, Qiong Yang, Qiaoling Zhu, Yuxiao Zou

**Affiliations:** 1 Sericultural & Agri-Food Research Institute, Guangdong Academy of Agricultural Sciences/Key Laboratory of Functional Foods, Ministry of Agriculture and Rural Affairs/Guangdong Key Laboratory of Agricultural Products Processing, Guangzhou 510610, China; snoopylen@126.com (E.L.);; 2 College of Food Science, South China Agricultural University, Guangzhou 510642, China

**Keywords:** *Ramulus mori* oligosaccharides, *Streptococcus mutans*, anti-caries, antibacterial, biofilm

## Abstract

*Ramulus mori* has been widely used in traditional Chinese medicine because of its physiological activities, including antibacterial, anti-inflammatory, and antioxidant activities. Antimicrobial properties of *Ramulus mori* extract have been well described. However, no information is available regarding on *Ramulus mori* oligosaccharides (RMOS). The aim of this study was to investigate the effects of RMOS on the growth and virulence properties of the cariogenic bacterium *Streptococcus mutans.* The effects of RMOS on the biofilm structure and virulence gene expression of *S. mutans* were also evaluated, and the results were compared with the effects of commercial prebiotic galactooligosaccharides. RMOS were found to have an antibacterial effect against *S. mutans*, resulting in significant reductions in acid production, lactate dehydrogenase activity, adhesion, insoluble extracellular polysaccharide production, glucosyltransferase activity, and biofilm formation in a dose-dependent manner. Moreover, the biofilm structure was visibly damaged. A quantitative real-time PCR assay revealed downregulation of virulence gene-regulated acid production, polysaccharide production, adhesion, biofilm formation, and quorum sensing. These findings suggest that RMOS may be a promising natural product for the prevention of dental caries.

## 1. Introduction

Caries is the most common disease of the oral cavity. It is a combination of many factors that lead to chronic and progressive damage to the hard tissues of the teeth [1]. It is generally believed that *Streptococcus mutans* (*S. mutans*) is crucial to the emergence and progression of dental caries [2]. Glucosyltransferase (GTF) is secreted by *S. mutans* and synthesizes glucans from sucrose, such as insoluble extracellular polysaccharides (IEPS), which mediates the adherence of *S. mutans* to the tooth surface and contributes to the formation of biofilm [3]. Therefore, an anaerobic and acidic environment is created, and carbohydrates are fermented to produce organic acids, which cause the teeth’s hydroxylapatite crystal structure to dissolve calcium and phosphate. This eventually leads to tooth demineralization and the formation of dental caries [4,5].

The options for preventing dental caries predominantly involve antibiotics, fluoride, and pit and fissure sealing [6]. However, as a result of the emergence of fluoride- and drug-resistant strains, cytotoxicity, and the constrained applicability spectrum of present preventative measures [7], research into natural, non-toxic medicines with anti-caries properties has gained increasing attention. To date, the anti-caries effects of a number of natural medicines have been confirmed. For example, it has been documented that reuterin and catechin work together synergistically to prevent *S. mutans* from growing and forming biofilms [8]. Nidus Vespae extract and its chemical fractions significantly inhibited the activity of cell-associated and extracellular GTF from *S. mutans* at sub-minimum inhibitory concentrations (MICs) [9]. Sodium new houttuyfonate downregulated expression of the *gtfBCD* and *comDE* gene systems and exhibited a synergistic effect with chlorhexidine [10].

The traditional Chinese medicine, *Ramulus mori*, which is extracted from the branches of the mulberry tree, contains active ingredients such as flavonoids, polysaccharides, alkaloids, and amino acids [11,12]. Polysaccharides are the main active substance of *Ramulus mori* and are composed of monosaccharides such as galactose, mannose, rhamnose, glucuronic acid, glucose, xylose, and arabinose [13]. *Ramulus mori* polysaccharides have pharmacological effects such as antibacterial and anti-inflammatory effects, as well as activities that relieve diabetes, protect the liver, and improve immunity [14,15,16]. Enzymatic degradation of polysaccharides reduces their molecular weight and improves their physiological activities and potential clinical applications. The antibacterial activity of oat β-glucan, housefly larvae chitooligosaccharides, and chitosanoligosaccharides gradually increased with the decrease in molecular weight [17,18]. To date, many studies have shown that functional oligosaccharides have good inhibitory effects on the growth of pathogenic microorganisms. Isomaltooligosaccharides cannot be utilized by *S. mutans* and thereby reduce acid production, and panose, specifically, and can effectively inhibit the formation of tartar [19]. Galactooligosaccharides (GOS) in breast milk protect the oral health of infants by inhibiting the adhesion of *S. mutans* [20]. Yu et al. confirmed that *Ramulus mori* polysaccharides inhibited the proliferation of *Escherichia coli*, *Staphylococcus aureus*, and *Pseudomonas aeruginosa* [13]. Based on this premise, our previous research found that *Ramulus mori* oligosaccharides (RMOS) were superior to *Ramulus mori* polysaccharides at inhibiting *S. mutans*.

The aim of this study was to investigate the effects of RMOS on the growth, virulence properties (adhesion, biofilm formation, acid production, dextran production, and adherence), and virulence gene expression of *S. mutans*. The findings supply a theoretical basis for utilizing RMOS in daily chemical oral care products.

## 2. Materials and Methods

### 2.1. Preparation of RMOS

*Ramulus mori* polysaccharide solution was prepared according to previous methods [21] with some modifications. Soak the *Ramulus mori* in a water bath at 80 °C for 4 h (1:4, *w*/*v*). Concentrate the supernatant to 1/4 of its original volume by vacuum filtration at 50 °C, and then precipitate with four volumes of 95% (*v*/*v*) ethanol at 4 °C for 24 h. The crude polysaccharide was obtained by centrifugal separation (9000× *g* for 15 min). Phenol-sulfuric acid was used to determine the polysaccharide content. For the production of the enzymatic hydrolysate containing *Ramulus mori* oligosaccharides (RMOS), *Ramulus mori* polysaccharide (3 mg/mL) was incubated with 485 U/mL *β*-glucanase (Yuanye Co., Ltd., Shanghai, China) at 45 °C for 4 h, and then lyophilized.

### 2.2. Bacterial Strain and Culture Conditions

*Streptococcus mutans* (ATCC 25175) was purchased from GDMCC (Guangzhou, China). The strain was cultured in BHI medium at 37 °C in an anaerobic incubator (80% N_2_, 10% H_2_, 10% CO_2_) for 24 h.

### 2.3. Minimum Inhibitory Concentration (MIC) and Minimum Bactericidal Concentration (MBC) Assays

The RMOS of twofold serial dilutions (192–0.75 mg/mL) was dissolved in medium added with 1% (*w*/*v*) sucrose. *S. mutans* (1% (*v*/*v*), 5 × 10^5^ CFU/mL) was cultured in an anaerobic incubator at 37 °C for 24 h. The negative control was *S. mutans* incubated without RMOS, while the blank control was *S. mutans*-free BHI media. The MIC was the lowest concentration of RMOS that inhibited visible *S. mutans* growth completely. A 20 µL sample was taken from the test tube with a concentration higher than the MIC and plated for colony enumeration. The MBC was the lowest concentration of RMOS that inhibited *S. mutans* growth completely.

### 2.4. Growth Curve Assay

Growth curve measurement was performed to determine the sensibility of bacteria to different concentrations of RMOS. GOS was utilized as a positive bacteriostatic control group, because the beneficial impact upon decreasing the adherence and growth of bacterial are well proved [20]. First, RMOS ranging from 1/8 MIC to the MIC were dissolved in BHI medium supplemented with 1% (*w*/*v*) sucrose. Then, 1% (*v*/*v*) of *S. mutans* was inoculated into the culture medium and cultured anaerobically for 24 h at 37 °C. Bacteria incubated without RMOS were used as a negative control, and culture BHI medium with an MIC of GOS was used as a positive control. Growth optical density of *S. mutans* measured at 630 nm with Multiskan spectroscopy at 2 h intervals (Spectra Max i3x, Molecular Devices, San Jose, CA, USA).

### 2.5. Glycolytic pH Drop Assay

The effect of different concentrations of RMOS on acid production by *S. mutans* was evaluated by a pH drop assay and acidity was measured as described previously [22]. *S. mutans* was inoculated into the culture medium and incubated anaerobically at 37 °C for 24 h. Bacteria incubated without RMOS served as a negative control, and culture medium with an MIC of GOS served as a positive control. The initial pH of each test tube was adjusted to 7.4 using 0.1 M KOH. After incubation, the pH of the culture supernatant was determined. The ΔpH value was calculated as the difference between the initial pH and the final pH.

### 2.6. Lactate Dehydrogenase (LDH) Measurement

The method of reducing coenzyme I oxidation was used to determine the activity of LDH [23]. After incubation, bacterial precipitates were collected and washed with PBS three times. After resuspending the cell pellet in 1 mL of Tris-HCl buffer containing 5 mg/mL lysozyme, the cells were incubated at 37 °C for 1 h. Bacterial cells were then disrupted by instantaneous ultrasound several times in an ice bath. After centrifugation for 20 min, the supernatants were collected and LDH activity was measured with a lactate dehydrogenase assay kit (Nanjing Jiancheng Bioengineering Company, Nanjing, China) following the manufacturer’s instructions.

### 2.7. IEPS Measurement 

Insoluble extracellular polysaccharides (IEPS) production was quantified as described previously [24], with some modifications. RMOS ranging from 1/8 MIC to the MIC were dissolved in BHI medium supplemented with 1% (*w*/*v*) sucrose. *S. mutans* was anaerobically inoculated into the culture medium. After incubation, the precipitations were collected and rinsed with PBS and 0.4 M NaOH three times. IEPS was formed by combining the supernatants. The IEPS were then precipitated with three times the volume of ethanol. After storage at 4 °C for 12 h, centrifugation, and resuspension in 0.4 M NaOH, the contents of IEPS were determined by the phenol sulfuric acid method.

### 2.8. GTF Measurement

Senpuku techniques were used to assess the effects of RMOS on the glucosyltransferase (GTF) activity of *S. mutans* in vitro [24]. Following 24 h incubation, three PBS washes were performed after collecting the bacterial precipitates. Solid ammonium sulfate was then added to the supernatant to 60% saturation, and the mixture was stored at 4 °C overnight. Following centrifugation, the GTF was collected, dissolved in 0.01 M PBS, then dialyzed until no ammonia was produced. A BCA kit (Beyotime Biotechnology Company, Shanghai, China) was used to determine the protein concentration of GTF. A substrate solution comprising 1 mL 0.2 M PBS, 1 mL 0.5 M sucrose, and 4 mL double-distilled water was prepared, to which the GTF preparation was added, and then incubated at 37 °C for 1 h. To stop the reaction, 50 µL of 0.24 M HCl was added. In order to separate the mixture, 1.25 mL of double-distilled water was centrifuged for 10 min. After centrifugation, the supernatant was collected to determine the amount of reducing sugar by the phenol–sulfuric acid method. GTF activity units (IU) were defined as follows: the amount of enzyme required to release 1 µmol reducing sugar from sucrose per minute under standard conditions (i.e., 1 h reaction at 37 °C). The GTF specific activity was calculated as follows:

Enzyme activity = amount of reducing sugar/(molecular weight of glucose × 60);

Relative activity = enzyme activity (mIU)/total protein (mg).

### 2.9. Adhesion Assay

Adhesion of *S. mutans* strains to a glass surface was achieved as previously described [25]. Bacteria were anaerobically incubated at a 30° angle with RMOS and GOS at 1/8 MIC to the MIC at 37 °C for 24 h. After culture, bacterial suspensions were transferred into a fresh tube, which was then washed with potassium PBS (0.05 mol/L, pH = 7.0) and the loosely adhered *S. mutans* was transferred to another new tube. This test tube was rinsed with PBS again to measure the number of *S. mutans* adhered to the tube wall. All tubes were centrifuged, and the precipitates were gathered and re-suspended in PBS. Absorbance was measured at 630 nm. Calculate the adhesion inhibition rate using the following formula:

$$Adhesion\;inhibition\;rate\left(\%\right)=\frac{adhesion\;rate\;of\;control\;group-adhesion\;rate\;of\;experimental\;group}{adhesion\;rate\;of\;control\;group}\times 100\%$$

### 2.10. Biofilm Formation Assay

The impact of RMOS on biofilm formation by *S. mutans* was investigated with the crystal violet method [26]. Briefly, *S. mutans* was inoculated into culture medium in a 96-well plate and cultured anaerobically for 24 h to allow for the formation of biofilm. After incubation, the spent medium and unattached bacteria were removed, then three washes with 200 µL of PBS were performed to remove planktonic bacteria. The biofilm was fixed by adding 100 µL of methanol and incubating for 15 min, followed by drying at 25 °C. The biofilm was then stained by adding 100 µL of 0.1% crystal violet to the wells for 5 min at room temperature. The wells were rinsed thoroughly with PBS until the control well appeared colorless and were then dried at room temperature. When dry, 100 µL of 95% (*v*/*v*) ethanol was added and the plates were placed at 37 °C for 30 min to dissolve the crystal violet. The inhibition of biofilm formation was determined using the following equation to measure the absorbance at 550 nm:

Inhibition rate of biofilm formation(%)=(1−experimental group/control group)×100%

### 2.11. Scanning Electron Microscopy (SEM)

*S. mutans* was cultivated with RMOS for 24 h in anaerobic conditions at 37 °C in media under a sterile cover slide. Cover slips was gently washed three times with PBS to remove loose bacteria, then fixed with 2.5% (*v*/*v*) glutaraldehyde for 120 min and dehydrated in a graded alcohol series (20%, 40%, 60%, 80%, and 100% ethanol). The cover slip was then coated with gold sputtering in a critical point drying vacuum. The bacterial morphology and adhesion were examined and imaged using SEM (S-3400N, Hitachi, Japan).

### 2.12. Confocal Laser Scanning Microscopy (CLSM)

A clean sterile cover slide was added to the wells of a 6-well plate and inoculated according to the above conditions. After anaerobic incubation at 37 °C for 24 h, the cover slide was washed with PBS gently three times to remove any non-adhered bacteria. *S. mutans* biofilms were stained with 5 µM Syto 9 and 30 µM propidium iodide (PI; Molecular Probes, Eugene, OR, USA) in the dark for 10 min before being imaged by a confocal microscope (Zeiss LSM710, Carl Zeiss, Oberkochen, Germany). A range of 490–635 nm for PI (dead bacteria) and 480–500 nm for Syto 9 (live bacteria) were set to the image collection gates. Each sample was randomly selected with five fields of view. Z sections were collected, and the biofilms were analyzed using the analysis software ZEN.

### 2.13. qRT-PCR Assay

The expression of virulence genes of *S. mutans,* including *ldh*, *gtfB*, *gtfC*, *gtfD*, *ftf, spaP*, *srtA*, *brpA. gbpB, luxs*, *comD*, and *comE*, was determined using a qRT-PCR assay. Anaerobic cultivation of *S. mutans* using RMOS and GOS. After incubation for 24 h, the bacteria were harvested using an RNA TRIzol kit (Tiangen, Beijing, China) by centrifugation, followed by total RNA extraction. A reverse transcription kit (TaKaRa, Bio Inc., Dalian, China) was used to reverse transcribe the RNA into cDNA. 16S RNA was used as an internal standard for amplification to normalize gene expression. NCBI Primer-BLAST software was used to design the optimal primers, as shown in Table 1. qRT-PCR was conducted using the ABI7300 drop digital PCR system (Applied Biosystems, Foster City, CA, USA) and SYBR Green Premix Ex Taq (TaKaRa, Kusatsu, Japan). The 25 µL reaction mixture comprised 2 µL of cDNA, 1 µL each of the upstream and downstream primers, 12.5 µL of SYBR Green Mix, and 8.5 µL of ddH_2_O. The amplification conditions were 95 °C activation for 3 min, then 40 cycles of denaturation at 95 °C for 5 s and annealing at 62 °C for 30 s, followed by extension at 72 °C for 30 s, and fluorescence data collection. Each sample was equipped with a virulence gene detection tube and an internal reference gene (16S rRNA) detection tube. The results were analyzed by the 2^−ΔΔCT^ method.

### 2.14. Analysis and Statistics

Treatments were carried out in triplicate for each experiment, and data are expressed as mean standard deviation (SD). Statistical analysis was determined by one-way ANOVA using SPSS 22.0, Origin 2019, and GraphPad Prism 8.1. *p* < 0.05 was considered significant and no significant difference was indicated by the same lowercase letter indicates (*p* > 0.05).

## 3. Results and Discussion

### 3.1. Antibacterial Activity of RMOS on S. mutans

Dental caries occurs as a result of the growth and reproduction of bacteria. Our findings revealed that RMOS have an antibacterial effect on *S. mutans* with a MIC = 24 mg/mL and a MBC = 48 mg/mL.

**Figure 1:**
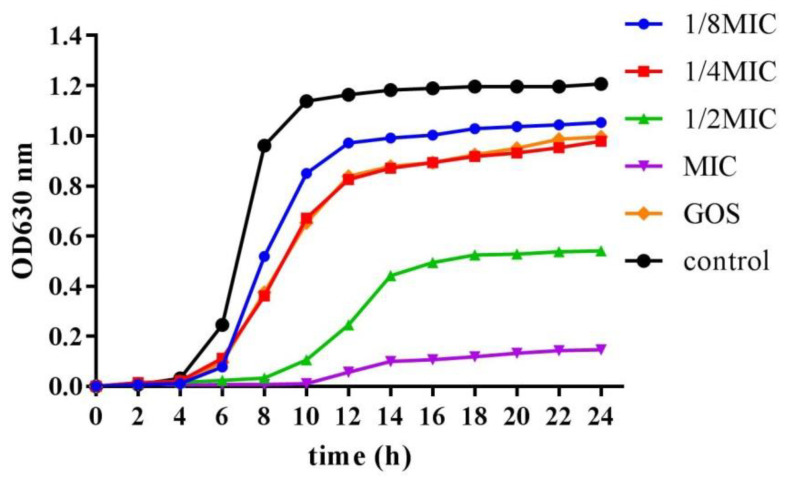
Growth curve of *S. mutans* with different concentrations of RMOS. RMOS at 1/8 MIC (3.00 mg/mL); 1/4 MIC (6.00 mg/mL); 1/2 MIC (12.00 mg/mL), and the MIC (24.00 mg/mL) for 24 h incubation. Bacteria incubated without RMOS served as a negative control, and a culture medium with the MIC (24.00 mg/mL) of GOS served as a positive control. OD = optical density.

Figure 1 shows the growth curves of planktonic cells of *S. mutans* after 24 h treatment with RMOS. RMOS ranging from 1/8 MIC to the MIC all exhibited an antibacterial effect on *S. mutans* and the inhibitory effect was obvious in the logarithmic phase of bacterial growth. The growth curve with GOS was similar to that observed with 1/4 MIC. It had no obvious effect on the growth curve at the MIC, while 1/8 MIC and 1/4 MIC showed relatively attenuated inhibition, demonstrating that the inhibitory effect of RMOS on *S. mutans* was dose-dependent. RMOS delayed the transition of bacteria growth to the logarithmic period. The drug-free group entered a growth plateau at 10 h, compared with 12 to 14 h at 1/8 MIC to 1/2 MIC. These results were consistent with the reported inhibition of the growth of planktonic *S. mutans* strains by low concentrations of xylitol [27].

### 3.2. RMOS Inhibits the Acidogenicity of S. mutans

**Figure 2:**
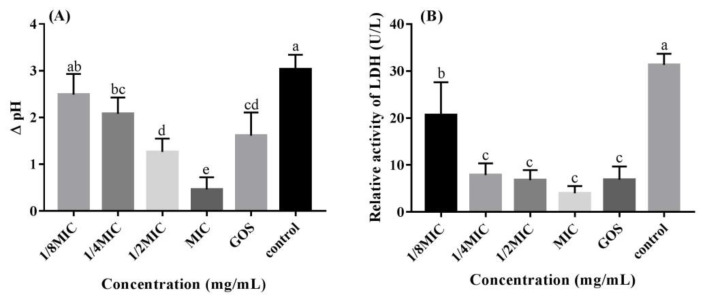
Effects of RMOS on the acidogenicity of *S. mutans*. (**A**) Glycolytic pH drop of *S. mutans*. (**B**) Relative activity of LDH of *S. mutans*. *S. mutans* was inoculated with RMOS at 1/8 MIC (3.00 mg/mL); 1/4 MIC (6.00 mg/mL); 1/2 MIC (12.00 mg/mL), and the MIC (24.00 mg/mL) and cultured anaerobically for 24 h. Bacteria incubated without RMOS served as a negative control, and a culture medium with the MIC (24.00 mg/mL) of GOS served as a positive control. Data are presented as means ± SD over three repetitions. Bars with various letters differ significantly (*p* < 0.05).

The impact of RMOS on the acidogenicity of *S. mutans* was investigated by testing the pH value of glycolysis of the bacterial cultures and examining the decrease in LDH activity. As presented in Figure 2, RMOS reduced the glycolytic pH of *S. mutans*. Further experiments demonstrated that the LDH of *S. mutans* was significantly reduced compared to the controls (*p* < 0.05). The inhibitory effect of GOS was similar to that of RMOS at 1/2 MIC. Carbohydrates are fermented by *S. mutans* to produce acid, which is the direct cause of dental caries. When the pH in dental plaque is lower than 5.5, the balance between demineralization and remineralization is broken, and dental caries will occur [28]. LDH is the main anaerobic metabolic pathway of glucose, and the key enzyme for the synthesis of lactic acid. Inhibiting the activity of LDH can reduce the production of lactic acid, thereby reducing the occurrence of dental caries [23]. Compared with sucrose alone, D-Tagatose delayed the decline of pH of medium [29]. Another report demonstrated that ellagic acid and quercetin reduced the glycolytic drop in pH and lactate production, but not lactate dehydrogenase activity [30]. However, Gj-CATH2 has an inhibitory effect on the expression of LDH at the enzymatic and transcriptional level but has no effect on the decrease in pH by bacterial glycolysis [22]. *S. mutans*’s glycolytic pH drop was inhibited by RMOS in a dose-dependent manner, and LDH activity was effectively reduced. The mechanism may be that a reduction in LDH activity affects the normal glycolysis process of bacteria, reducing acid production, and thereby reducing the risk of dental caries.

### 3.3. RMOS Inhibits IEPS and the GTF Activity of S. mutans

**Figure 3:**
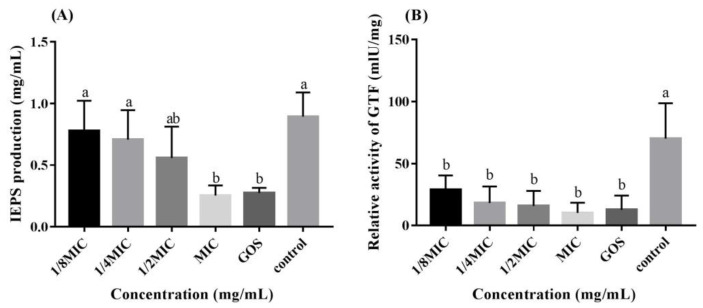
Effects of RMOS on the synthesis of IEPS of *S. mutans*. (**A**) IEPS production by *S. mutans*. (**B**) Relative activity of GTF of *S. mutans*. *S. mutans* was inoculated with RMOS at 1/8 MIC (3.00 mg/mL); 1/4 MIC (6.00 mg/mL); 1/2 MIC (12.00 mg/mL), and the MIC (24.00 mg/mL) and anaerobically cultured for 24 h. Bacteria incubated without RMOS served as a negative control, and culture medium with the MIC (24.00 mg/mL) of GOS served as a positive control. Data are presented as means ± SD over three repetitions. Bars with various letters differ significantly (*p* < 0.05).

We found that RMOS treatment decreased the production of IEPS and decreased the GTF activity of *S. mutans*, as demonstrated by assays of IEPS synthesis and GTF activity. As indicated in Figure 3A, IEPS synthesis decreased in the experimental groups as the RMOS concentration increased. At the MIC and for the control, IEPS synthesis was remarkably reduced (*p* < 0.05). GTF activity was significantly inhibited by RMOS treatment (*p* < 0.05), as presented in Figure 3B. GTF activity and IEPS showed a positive correlation. *S. mutans* uses carbohydrates to synthesize IEPS to promote acid production, adhesion, and aggregation [31]. GTF is an enzyme for glucose metabolism secreted by *S. mutans*, which can polymerize sucrose into α-1,3- and α-1,6-linked glucans, including water-soluble glucans and water-insoluble glucans [32]. IEPS is the basic skeleton structure of the biofilm. It can mediate sucrose-dependent adhesion and the aggregation of bacteria, promote their colonization, and accelerate the formation of dense biofilms [33]. Theaflavins were found to suppress GTF activity and the synthesis of glucan-binding proteins, which decreased *S. mutans’* ability to form biofilms [34]. Therefore, we speculate that RMOS inhibit the synthesis of IEPS by targeting and inhibiting GTF activity, thereby reducing the matrix in the biofilm and reducing the density of the biofilm, ultimately affecting the normal structure of the biofilm.

### 3.4. RMOS Suppresses Biofilm Formation by S. mutans

**Figure 4:**
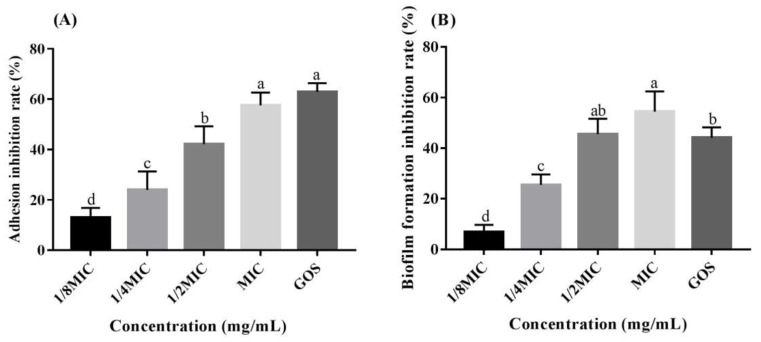
Effects of RMOS on the biofilm of *S. mutans*. (**A**) Adhesion of *S. mutans*. (**B**) Formation of *S. mutans* biofilm. *S. mutans* was inoculated with RMOS at 1/8 MIC (3.00 mg/mL), 1/4 MIC (6.00 mg/mL), 1/2 MIC (12.00 mg/mL), and the MIC (24.00 mg/mL) and cultured anaerobically for 24 h. Bacteria incubated without RMOS served as a negative control, and a culture medium with the MIC (24.00 mg/mL) of GOS served as a positive control. Data are expressed as means ± SD of three repeats. Bars with different letters differ significantly (*p* < 0.05).

The effect of RMOS on *S. mutans* adhesion to a glass surface was evaluated (Figure 4A). Inhibition of adhesion rates of 12.97%, 23.93%, 42.16%, 57.55%, and 62.93% were detected for 1/8 MIC, 1/4 MIC, 1/2 MIC, and the MIC of GMOS and GOS, respectively. Different concentrations of RMOS and GOS treatment therefore decreased the adhesion of *S. mutans*. The inhibitory impact correlated with the concentration, and the discrepancy between the different concentrations was significant (*p* < 0.05). At the MIC, RMOS and GOS could inhibit more than 50% of *S. mutans* from adhering to the test tube. The adhesion ability of bacteria is closely related to their ability to cause caries. Bacteria in the oral cavity are reported to undergo sucrose-dependent, extracellular glycan production-mediated adherence [35] and the adhesion of *S. mutans* to the tooth surface is an important factor in the occurrence of dental caries [36]. Inhibiting bacteria adhesion within the oral cavity is one effective method of preventing dental caries. The effect of licorice isoflavans was reported to attenuate the adhesion of *S. mutans* to a saliva-coated hydroxylapatite surface [37]. Furthermore, it was reported that GOS and lactose decreased the exopolysaccharide-mediated adhesion of *S. mutans* to a glass surface [20]. Our results were consistent with these findings, indicating that RMOS inhibits *S. mutans* adherence to hard surfaces.

The biofilm biomass of bacteria exposed to RMOS and GOS was measured by a crystal violet assay, and the results are displayed in Figure 4B. RMOS dose-dependently and significantly reduced the biofilm formation of *S. mutans*. There was a 6.87%, 25.48%, 45.55%, 54.44%, and 44.13% reduction at 1/8 MIC, 1/4 MIC, 1/2 MIC, and the MIC of RMOS and GOS respectively, relative to the control group. There was no significant difference between 1/2 MIC for RMOS and GOS (*p* > 0.05). The biofilm produced by *S. mutans* is composed of extracellular polysaccharides as the main matrix component and is affected by many factors, such as automatic cell aggregation and co-aggregation, extracellular polymer synthesis, and virulence gene expression [38]. A number of studies have evaluated the biofilm-inhibiting properties of natural anti-caries drugs. For example, Licorice extract had no effect on the removal of mature biofilm, but it could partially penetrate the biofilm and eliminate bacterial cells [39]. Baicalein remarkably reduced the ability of biofilm formation by *S. mutans* without growth inhibition [36]. By targeting the adhesion and biofilm maturation stages, Gj-CATH2 inhibited the biofilm formation of *S. mutans* [22], which was consistent with our results.

### 3.5. RMOS Destroys the Biofilm Structures of S. mutans

**Figure 5:**
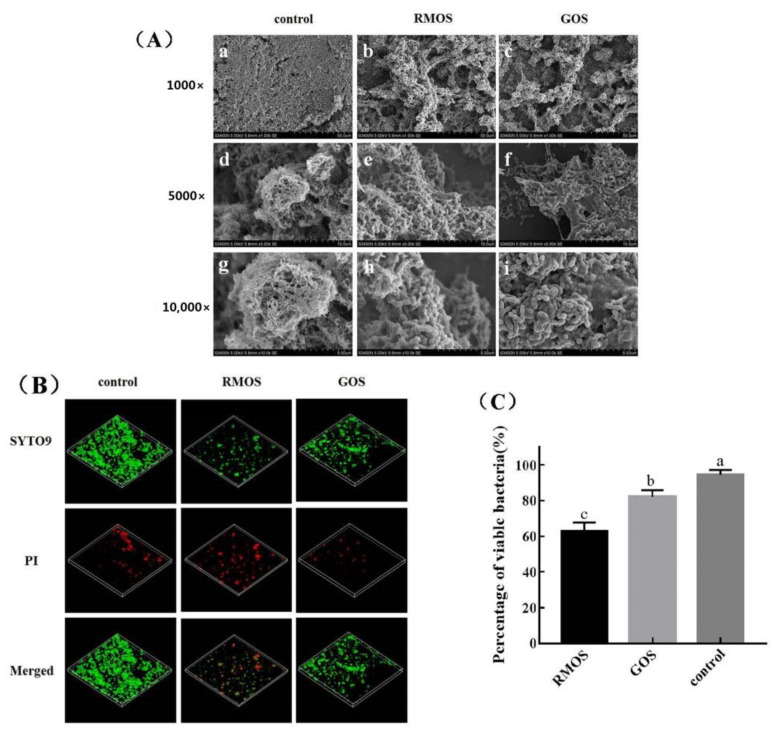
Effects of RMOS on biofilm structures of *S. mutans*. (**A**) SEM images of *S. mutans*. (**B**) CLSM images of *S. mutans*. (**C**) The percentage of viable bacteria in the biofilm. *S. mutans* was inoculated with RMOS at the MIC (24.00 mg/mL) and anaerobically cultured for 24 h. Bacteria incubated without RMOS served as a negative control, and a culture medium with the MIC (24.00 mg/mL) of GOS served as a positive control. The microstructure of the *S. mutans* biofilm on glass coverslips was observed at 1000×, 5000×, and 10,000× magnification by SEM. Biofilms were stained by STYO9 and PI and observed by CLSM. Live *S. mutans* was marked in green, and dead *S. mutans* was marked in red. Three separate experiments’ representative data are shown in the photographs. Data are expressed as means ± SD of three repeats. Bars with different letters differ significantly (*p* < 0.05).

The biofilm structure of *S. mutans* was observed by SEM, and structural changes were observed after RMOS treatment (Figure 5A). The biofilm structure of *S. mutans* showed obvious morphological changes under SEM after treatment with RMOS and GOS at the MIC. In comparison to the control group, the quantity of *S. mutans* decreased following treatment with RMOS and GOS. A small number of bacteria adhered, and the adhesion density was sparse with relatively few bacterial agglomerates. The *S. mutans* biofilm was observed under CLSM, and the results are displayed in Figure 5B. Among those in the control group, a large green area of aggregates (live bacteria) was observed compared with few red cells (dead bacteria), and the percentage of live bacteria was 94.48% ± 2.25%. This confirmed strong bacterial activity and a relatively intact biofilm in the absence of drugs. After RMOS treatment, the number of green viable bacteria and aggregates decreased, whereas the number of red dead bacteria increased, and the percentage of viable bacteria in the biofilm was only 62.78% ± 4.05%, which was significantly lower than the control group (*p* < 0.05). It was found that RMOS can restrain the proliferation and adhesion of *S. mutans*, reduce the number of viable bacteria, destroy the morphology of bacterial biofilm, and reduce further biofilm formation.

### 3.6. RMOS Downregulates the Expression of Virulence Genes of S. mutans

**Figure 6:**
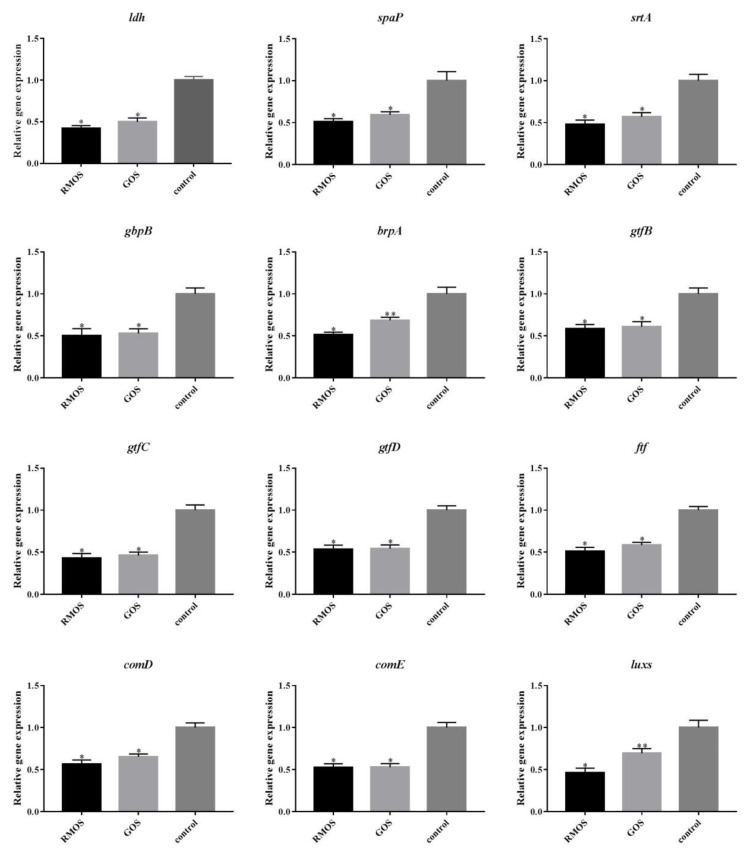
Effects of RMOS on the expression of virulence genes of *S. mutans. S. mutans* was inoculated with RMOS at the MIC (24.00 mg/mL) and anaerobically cultured for 24 h. Bacteria incubated without RMOS served as a negative control, and culture medium with the MIC (24.00 mg/mL) of GOS served as a positive control. Data are expressed as means ± SD of three repeats. Statistical significance is identified as * *p* < 0.05, ** *p* < 0.01.

Figure 6 displays the impact of RMOS on the expression of virulence genes in *S. mutans*. The results demonstrated that treatment with RMOS and GOS downregulated the expression of cariogenic genes of *S. mutans*, such as *ldh*, *gbpB*, *brpA*, *spaP*, *srtA*, *gtfB*, *gtfC*, *gtfD*, *ftf*, *comD*, *comE*, and *luxs*, compared with the control. The downregulation of expression of *luxs* and *brpA* by RMOS was significant compared with GOS (*p* < 0.05).

The cariogenic characteristics of *S. mutans* are regulated by several genes, which are involved in acid production (*ldh*), glucan production (*gtfB*, *gtfC*, *gtfD*, ftf), adhesion and biofilm formation (*brpA*, *spaP*, *gbpB*, *strA*), and quorum sensing (*comD*, *comE*, *luxs*). Lactate dehydrogenase catalyzes the conversion of pyruvate to lactic acid, which is encoded by *ldh* [40]. The decrease in *ldh* expression could explain the decrease in LDH activity and the inhibition of acid production.

The genes *gtfB*, *gtfC*, and *gtfD* encode for GTF. GTF is involved in the synthesis of extracellular polysaccharides, mediates bacterial adhesion, and enhances the structural integrity of biofilms [41]. Fructan transferase (Ftf), encoded by the *ftf* gene, promotes the synthesis of fructans by *S. mutans*, increases the aggregation of bacteria, and contributes to the formation of biofilm. RMOS downregulated the expression of *gtfB*, *gtfC*, *gtfD*, and *ftf*, thereby reducing the production of IEPS, which contributed to the inhibition of bacterial adhesion and the formation of biofilm. This also explains the SEM data.

During the initial adhesion process of *S. mutans*, the surface adhesion protein encoded by *spaP* is crucial [42]. The downregulation of *brpA* might affect the expression of *spaP* and *srtA*, which contributes to biofilm formation by *S. mutans*. *Gbpb* encodes the glucan binding protein GbpB, which mediates the adhesion of *S. mutans* to glucan, and plays an important role in the sucrose-dependent adhesion of *S. mutans* [43]. The differences in the expression of these genes indicated that the regulation of these proteins may play a role in RMOS inhibiting the initial formation of *S. mutans* biofilm.

Quorum-sensing systems control gene expression and regulate bacterial population density. For example, the intra specific density sensing signal is adjusted by the ComDE system, and the inter species density sensing signal is adjusted by the Luxs system. *ComD* encodes the histidine kinase receptor and reacts to the ability stimulating peptide (CSP); COME encodes intracellular response regulatory factors and mediates the expression of downstream genes. Histidine kinase receptor was encoded by *ComD*. *ComD* could show a response to stimulating peptide (CSP).

Intracellular response regulator was encoded by *comE*. *comE* could mediate downstream gene expression. S-ribose homocysteine lyase (*luxS*), which is mainly involved in the compactness of biofilm structures, is encoded by the *luxS* gene [44]. These factors mediate the stress response and the production of virulence factors to strengthen the adaptive capacity and disease-causing ability of *S. mutans* [8]. Reducing the expression of *comD*, *comE*, and *luxs* may aid the inhibition of bacterial growth and resistance to acidic environments.

Overall, RMOS appears to suppress *S. mutans* by modulating the expression of related genes. Downregulation of the expression of related virulence genes may explain the reduction in the drop in glycolytic pH, IEPS production, biofilm formation, and the biofilm structure damage.

## 4. Conclusions

Our findings confirmed that RMOS had effects on multiple steps during the cariogenic process of *S. mutans*, thereby effectively preventing the occurrence of dental caries. Future research will focus on incorporating RMOS into daily chemical products for oral care. In the current study, only biofilm formed by a single bacterium was considered. Further research will analyze effects on the formation, morphology, and metabolism of mixed biofilms.

## Figures and Tables

**Table 1:** Primers used in quantitative real-time PCR.

Name	Sequence (5′–3′)
*ldh*	F:ACTTCACTTGATACTGCTCGTT
	R:ACACCAGCTACATTGGCATGA
*gtfB*	F:ACGAACTTTGCCGTTATTGTCA
	R:AGCAATGCAGCCAATCTACAA
*gtfC*	F:CTCAACCAACCGCCACTGTT
	R:GGTTTAACGTCAAAATTAGCTGTATTAG
*gtfD*	F:TGTCTTGGTGGCCAGATAAAC
	R:GAACGGTTTGTGCAGCAAGG
*ftf*	F:CCTGCGACTTCATTACGATTGGTC
	R:ATTGGCGAACGGCGACTTACTC
*spaP*	F;TGGGATAGTTCAGATGCGCC
	R;AGGATCAGCAGGCACAACAA
*srtA*	F:TGGCAATTCCGCCAATTACAG
	R;AAGGCTGCCCATTCTTCCTT
*luxs*	F:GCTTTGATGACTGTGGCTATTTG
	R:ACTGCAGGCCTTCATACTATTG
*comD*	F:CGCGATTGGAGCCTTTAG
	R:CCTGAAATTCAGTTAGCCTTT
*Come*	F:GGCTACTTCCAGTCCTTTCTTT
	R:AACCACCATTGCAGCTATCA
*gbpB*	F:ATGGCGGTTATGGACACGTT
	R:TTTGGCCACCTTGAACACCT
*brpA*	F:GGAGGAGCTGCATCAGGATTC
	R:AACTCCAGCACATCCAGCAAG
16s RNA	F:CCTACGGGAGGCAGCAGTAG
	R:CAACAGAGCTTTACGATCCGAAA

## Data Availability

The data are available from the corresponding author.
